# Responsible Design, Integration, and Use of Generative AI in Mental Health

**DOI:** 10.2196/70439

**Published:** 2025-01-20

**Authors:** Oren Asman, John Torous, Amir Tal

**Affiliations:** 1 Department of Nursing, Faculty of Medical and Health Sciences, Tel Aviv University, P.O.B 39040, Ramat Aviv, Tel Aviv, 6997801, Israel, 972 547608020; 2 The Samueli Initiative for Responsible AI in Medicine, Tel Aviv University, Tel Aviv, Israel; 3 Department of Psychiatry, Beth Israel Deaconess Medical Center, Harvard Medical School, Cambridge, United States; 4 Faculty of Medical and Health Sciences, Tel Aviv University, Tel Aviv, Israel

**Keywords:** responsible AI in medicine, AI ethics, digital mental health ethics, artificial intelligence, large language model, model alignment

## Abstract

Generative artificial intelligence (GenAI) shows potential for personalized care, psychoeducation, and even crisis prediction in mental health, yet responsible use requires ethical consideration and deliberation and perhaps even governance. This is the first published theme issue focused on responsible GenAI in mental health. It brings together evidence and insights on GenAI’s capabilities, such as emotion recognition, therapy-session summarization, and risk assessment, while highlighting the sensitive nature of mental health data and the need for rigorous validation. Contributors discuss how bias, alignment with human values, transparency, and empathy must be carefully addressed to ensure ethically grounded, artificial intelligence–assisted care. By proposing conceptual frameworks; best practices; and regulatory approaches, including ethics of care and the preservation of socially important humanistic elements, this theme issue underscores that GenAI can complement, rather than replace, the vital role of human empathy in clinical settings. To achieve this, an ongoing collaboration between researchers, clinicians, policy makers, and technologists is essential.

## Introduction

The continued development of generative artificial intelligence (GenAI) and large language models (LLMs) shows potential in many fields, including high-stakes areas such as education, judicial work, security, and health. Utilizing this potential responsibly requires thoughtful deliberation and consideration and the creation of guidelines and conceptual frameworks that encompass the complexities of some of these fields.

The name of this theme issue reflects its focus—“Responsible Design, Integration, and Use of Generative AI in Mental Health.” The current abilities of GenAI models for language generation and image synthesis already demonstrate their ever-growing potential use in personalized mental health psychoeducation, diagnosis, treatment planning, and interventions. However, integrating any of these applications within the mental health care realm requires careful examination, given the sensitive nature of mental health data, research, and interventions and the various capacities that may be expected of these models in these realms, to be considered of acceptable professional standard. Recent studies highlight the significant ethical challenges posed by GenAI, emphasizing the need for robust governance frameworks to mitigate risks and enhance the trustworthiness of these technologies [1-3].

This theme issue unites diverse stakeholders in exploring and adding a critical building block for the global challenge of conceptualizing and operationalizing responsible GenAI in mental health. It includes a collection of articles that examine the advantages, challenges, and potential risks associated with deploying GenAI models in mental health care while also proposing guidelines and best practices for their ethical and responsible implementation. Several papers discuss the application of GenAI in clinical settings; the ethical implications of artificial intelligence (AI)–driven mental health interventions; and the development of new frameworks to ensure the alignment of GenAI systems with human values, virtues, and ethical standards. These include transparency, accountability, and fairness in AI applications; privacy and data security [4]; and authenticity and congruence [5].

The exploration of GenAI’s role in mental health is particularly timely, given its rapid adoption and the evolving landscape of digital health technologies. Recent research has highlighted the transformative potential of GenAI in creating personalized mental health interventions that can enhance care delivery and patient outcomes. For instance, GenAI models are already used to generate therapeutic content, simulate dialogues for therapy, and even predict mental health conditions based on language patterns and sentiment analysis [6]. However, this potential is accompanied by significant ethical and practical challenges, such as ensuring the accuracy and reliability of AI-generated content and preventing the misuse of these technologies [7]. This theme issue provides a platform for in-depth discussions on these topics and proposes actionable insights for the responsible integration of GenAI in mental health care.

## Current Capabilities and Limitations

We begin by exploring GenAI’s capabilities and limitations in mental health applications. Although the ever-evolving capacities explored in any research are, by definition, representatives of the time and models examined, the conceptual and normative-related discussions could have longer-term implications and relevance. The first paper, “Capacity of Generative AI to Interpret Human Emotions From Visual and Textual Data: Pilot Evaluation Study” [8], evaluates the ability of ChatGPT-4 and Google Bard to interpret human emotions from both visual and textual data. Using the Reading the Mind in the Eyes Test and the Levels of Emotional Awareness Scale, the study found that ChatGPT-4 performed well in both visual and textual emotion recognition, aligning closely with human standards. Google’s GenAI Bard, however, showed limitations in visual emotion interpretation. This paper emphasizes the need for inclusive data and stringent oversight to ensure accurate and reliable emotional recognition by AI systems.

The second paper, “Comparing the Perspectives of Generative AI, Mental Health Experts, and the General Public on Schizophrenia Recovery: Case Vignette Study” [9], compares the perspectives of GenAI models, mental health professionals, and the public on schizophrenia recovery. The findings show that some AI models align closely with professional views, while others, like ChatGPT-3.5, demonstrate pessimism that could negatively impact patient motivation. The study highlights the potential and limitations of AI in providing clinical prognoses and underscores the need for rigorous validation of AI applications in mental health.

The third paper, “Suicide Risk Assessments Through the Eyes of ChatGPT-3.5 Versus ChatGPT-4: Vignette Study” [10], examines the capability of ChatGPT models to assess suicide risk based on vignettes. The findings indicate that ChatGPT-4’s assessments align more closely with those of mental health professionals compared to ChatGPT-3.5, which often underestimates suicide risk. These findings highlight the potential of advanced AI models to support mental health professionals but also underscore the necessity for further research and careful implementation to ensure accurate and safe use in clinical settings.

The paper “Exploring the Efficacy of Large Language Models in Summarizing Mental Health Counseling Sessions: Benchmark Study” [11] evaluates the performance of state-of-the-art LLMs in summarizing therapy sessions. By introducing the Mental Health Counseling-Component–Guided Dialogue Summaries dataset and assessing task-specific LLMs, like MentalLlama, Mistral, and MentalBART, the study demonstrates their promise while emphasizing their current limitations in terms of clinical applicability. Expert assessments revealed the need for further refinement and validation before such tools can be integrated into practice.

Another key contribution, “Large Language Models Versus Expert Clinicians in Crisis Prediction Among Telemental Health Patients: Comparative Study” [12], compares GPT-4’s performance with that of senior clinicians in predicting suicide crises based on intake data. Although GPT-4 approached clinician-level performance in some metrics, its reliability was limited by sensitivity and bias issues. The study underscores the potential such tools have for augmenting crises prediction but highlights the need for additional safety measures and validation.

## Ethical and Humanistic Considerations

Herein, we delve into the ethical and humanistic considerations of GenAI in mental health.

In “Exploring Bias(es) of Large Language Models in the Field of Mental Health – A Comparative Study Investigating the Effect of Gender and Sexual Orientation in Anorexia Nervosa and Bulimia Nervosa Case Vignettes” [13], the authors showed that LLMs assigned lower mental health–related quality of life scores to men compared to women with a similar eating disorder severity, with no real-world epidemiological evidence for such a pattern. This may reflect historical underrepresentation and societal biases in the data used for training the model and raises questions about how such biases can be mitigated by users as well as developers.

Next, we address the ethical implications of humanizing AI and the importance of empathy in therapeutic contexts.

The paper “The Role of Humanization and Robustness of Large Language Models in Conversational Artificial Intelligence for Individuals With Depression: A Critical Analysis” [14] explores the use of LLMs, such as OpenAI’s ChatGPT-4, in mental health care. It highlights their potential to offer personalized therapeutic support for patients with depression through context-aware interactions. However, it also identifies significant ethical and technical challenges, including the risks of humanizing LLMs and their lack of contextualized robustness. Humanization can lead to unrealistic expectations and overtrust, while inadequate robustness may cause inconsistent and potentially harmful responses. The authors recommend clear communication of AI limitations, fine-tuning with high-quality data, and interdisciplinary research to responsibly integrate LLMs in mental health care, thereby enhancing patient support while minimizing risks.

The paper “The Machine Speaks: Conversational AI and the Importance of Effort to Relationships of Meaning” [15] explores the implications of using conversational AI in place of human effort in interpersonal relationships. The authors emphasize that effort in relationships conveys intrinsic value and meaning, which can be lost when machines take over these interactions. They discuss the importance of maintaining human effort in therapeutic contexts to preserve the meaningful engagement and personal growth that come from human-to-human interactions. This paper encourages a critical examination of the potential losses in meaning and opportunities for self-understanding when relying on GenAI.

Following this, the paper “Considering the Role of Human Empathy in AI-Driven Therapy” [16] addresses the critical role of empathy in therapy. It evaluates whether AI-driven therapy can replicate empathic interactions. The authors define different aspects of empathy, compare the empathic capabilities of humans and GenAI, and discuss when human empathy is most needed in therapeutic settings. They call for ongoing research and dialogue to ensure that AI-mediated therapy maintains the essential human element of empathy, which is crucial for effective therapeutic outcomes.

In “The Artificial Third: A Broad View of the Effects of Introducing Generative Artificial Intelligence on Psychotherapy” [17], the authors introduce the concept of the “artificial third” in psychotherapy, following Freud’s theory of narcissistic blows. They argue that GenAI represents a significant shift in how we perceive society, interrelationships, and self. They raise important questions about transparency, autonomy, and the irreplaceable human elements in therapy, suggesting that with ethical consideration, the artificial third can enhance but not replace the human touch in therapeutic relationships.

## GenAI Alignment With Values and Virtues

Finally, we consider the alignment of GenAI with human values and regulatory perspectives.

The study “Assessing the Alignment of Large Language Models With Human Values for Mental Health Integration: Cross-Sectional Study Using Schwartz’s Theory of Basic Values” [18] evaluates whether LLMs align with human values, using Schwartz’s theory of basic values. The authors found that while this framework can characterize value-like constructs within LLMs, there are significant divergences from human values, raising ethical concerns. They call for standardized alignment processes to ensure that LLMs are integrated into mental health care in a way that respects and reflects diverse human values.

Another important contribution is the article “Regulating AI in Mental Health: Ethics of Care Perspective” [19], which argues that the dominant responsible AI approach is insufficient because it overlooks the impact of AI on human relationships. The author proposes an ethics of care approach to AI regulation, which addresses AI’s impact on human relationships and establishes clear responsibilities for developers. They highlight the potential for emotional manipulation and the risks involved, proposing a series of considerations grounded in the ethics of care for developing AI-powered therapeutic tools.

Finally, the article “An Ethical Perspective on the Democratization of Mental Health With Generative AI” [20] explores the historical context of democratizing information and argues that GenAI technologies represent a new phase in this movement, offering improved accessibility to mental health knowledge and care. However, it also highlights the significant risks and challenges that need careful consideration. The paper proposes a strategic questionnaire for assessing AI-based mental health applications, advocating for an approach that is both ethically grounded and patient-centered.

## Conclusions

The papers comprising this special issue make essential and exciting contributions to the field of digital mental health, specifically focusing on the responsible integration and use of GenAI. These studies showcase the already remarkable abilities of LLMs and allude to the potential of integrating GenAI in mental health diagnosis, treatment, rehabilitation, and recovery while also raising awareness of technical, clinical, philosophical, and ethical challenges related to safety and efficacy.

This theme issue is merely one stepping stone that is part of an ongoing global effort. Responsible AI frameworks for mental health must be adapted and integrated into local and international governance frameworks, thereby acknowledging that the current extraordinary opportunity also presents a profound professional and societal challenge. By fostering ongoing dialogue and collaboration among researchers, clinicians, ethicists, policy makers, and technologists, we can harness the benefits of GenAI to enhance mental health care while upholding principles, values, and virtues fundamental to humanistic care.

Together, we can ensure that this technology serves as a tool for doing good, augmenting human capabilities while avoiding harm and respecting and retaining the socially important humanistic elements of empathy, authenticity, and connection.

## Abbreviations

AI: artificial intelligence
GenAI: generative artificial intelligence
LLM: large language model
